# Integrating single-molecule FRET and biomolecular simulations to study diverse interactions between nucleic acids and proteins

**DOI:** 10.1042/EBC20200022

**Published:** 2021-04-16

**Authors:** Joshua C. Sanders, Erik D. Holmstrom

**Affiliations:** 1 Department of Chemistry, University of Kansas, Lawrence, KS, U.S.A.; 2 Department of Molecular Biosciences, University of Kansas, Lawrence, KS, U.S.A.

**Keywords:** Conformational Dynamics, FRET, Nucleic Acids, Proteins, Simulations, Single-Molecule

## Abstract

The conformations of biological macromolecules are intimately related to their cellular functions. Conveniently, the well-characterized dipole–dipole distance-dependence of Förster resonance energy transfer (FRET) makes it possible to measure and monitor the nanoscale spatial dimensions of these conformations using fluorescence spectroscopy. For this reason, FRET is often used in conjunction with single-molecule detection to study a wide range of conformationally dynamic biochemical processes. Written for those not yet familiar with the subject, this review aims to introduce biochemists to the methodology associated with single-molecule FRET, with a particular emphasis on how it can be combined with biomolecular simulations to study diverse interactions between nucleic acids and proteins. In the first section, we highlight several conceptual and practical considerations related to this integrative approach. In the second section, we review a few recent research efforts wherein various combinations of single-molecule FRET and biomolecular simulations were used to study the structural and dynamic properties of biochemical systems involving different types of nucleic acids (e.g., DNA and RNA) and proteins (e.g., folded and disordered).

## Introduction

Spectroscopic methods are incredibly valuable for biochemists. For example, in an absorbance measurement the amount of light transmitted through a sample is used to quantify the optical density, and thus concentration, of biomolecular chromophores in solution [1]. Importantly, the absorption of certain wavelengths of light will temporarily excite these chromophores from a low energy ground state to a higher energy excited state. Fluorescence is a photophysical process that allows certain excited state chromophores (called fluorophores) to relax back down to the ground state, with some of the excess energy dissipated via the radiative emission of a photon.

**Figure 1:**
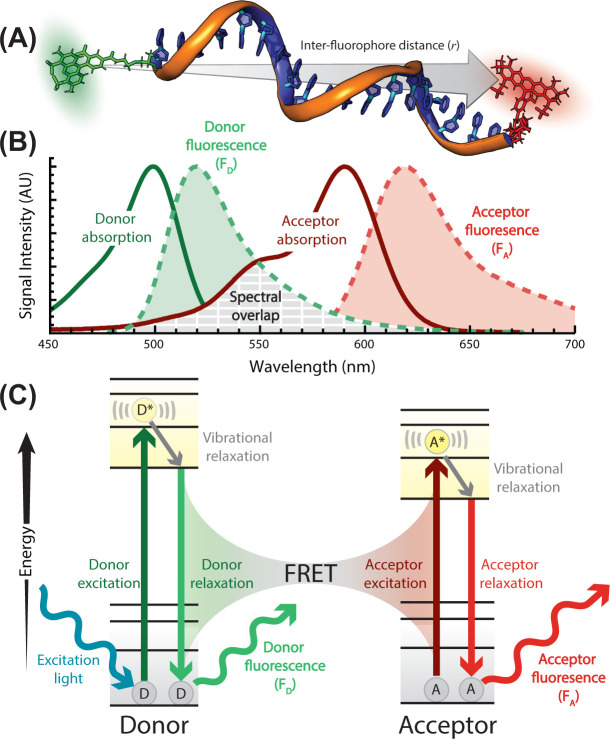
Fundamental principles of FRET (**A**) Computationally derived model of a 19 nt poly-dT single-stranded nucleic acid in an extended helical conformation at low ionic strength [20]. The DNA is labeled with a donor (i.e., Alexa Fluor 488, green) and acceptor (i.e., Alexa Fluor 594, red) fluorophore. (**B**) Absorption (solid) and emission (dashed) spectra of the specific donor (green) and acceptor (red) fluorophores depicted in (**A**), with the spectral overlap highlighted in gray. (**C**) Perrin-Jablonski energy-level diagram for fluorescence and FRET, highlighting the photophysical events associated with each process.

The ability to measure fluorescence can greatly enhance detection sensitivity [2], which makes fluorescence spectroscopy a particularly attractive choice when monitoring chemical reactions occurring in samples that contain trace amounts of biological molecules [3–5]. Although the components of some biological molecules are intrinsically fluorescent, many fluorescence-based approaches involve labeling biomolecules with a fluorophore (Figure 1A). Some key properties to consider when choosing a fluorophore include: the quantum yield, the extinction coefficient, and the absorption and emission spectra (Figure 1B). It is also important to consider the biocompatibility of fluorophores as they can often be quite hydrophobic or decorated with charged groups to increase solubility, both of which may facilitate potentially unwanted interactions with the biomolecules to which they will ultimately be coupled. Many of the most common fluorophores are sub-kilodalton highly substituted aromatic hydrocarbons [6,7]. Importantly, organic fluorophores are also relatively easy to derivatize for enhanced biocompatibility and facile conjugation chemistries, thereby enabling site-specific labeling of various functional groups in proteins [8–10] and synthetically modified nucleic acids [11–13]. Some of the most common bioconjugation methods utilize maleimide or N-hydroxysuccinimide moieties to couple these fluorophores to thiols and primary amines within the biomolecule of interest via flexible aliphatic linkers to minimize the likelihood for impaired biochemical function [14]. However, bioconjugation [15] and the local chemical environment associated with biological macromolecules [16] can alter the photophysical properties of the fluorophores. Therefore, one should be cautious when placing the fluorophore immediately adjacent to potential quenchers like tryptophan [17] or guanine residues [18], as well as other groups that lead to changes in the spectroscopic characteristics of the fluorophores [19].

## Principles of FRET

The utility of fluorescence-based spectroscopies can be further enhanced by introducing a second fluorophore into the system. Energetic coupling between the transition dipoles of these two fluorophores makes it possible for the excited ‘donor’ fluorophore (D*) to nonradiatively transfer energy to a nearby ground-state ‘acceptor’ fluorophore (A), yielding a donor fluorophore in the ground state (D) and an acceptor fluorophore in an excited state (A*). This photophysical phenomenon is famously known as Förster resonance energy transfer [21] or FRET (Figure 1C). The probability that an excited donor fluorophore returns to the ground state via FRET, which we will refer to as the transfer efficiency (*E*), depends on two key nanoscopic distances associated with the proximal fluorophores (eqn 1).

(1) $$E=\frac{R_{0}^{6}}{R_{0}^{6}+r^{6}}$$

The first key distance that influences the transfer efficiency is simply the physical distance, *r*, between the centers of the donor and acceptor fluorophores. The second key distance, *R*_0_, is the characteristic Förster distance, which is generally in the range of 2–7 nm [22,23] and depends on several important photophysical properties of the FRET pair (eqn 2).

(2) $$R_{0}\propto {\left(J\Theta_{D}n^{-4}\kappa^{2}\right)}^{1/6}$$

The overlap integral, *J*, characterizes the energetic resonance associated with the two coupled transition dipoles and is related to the spectral overlap of the normalized donor emission spectrum and the acceptor absorption spectrum (Figure 1B). Next, we have Θ*_D_*, which is the fluorescence quantum yield of the donor fluorophore in the absence of a nearby acceptor fluorophore, as well as *n*, which is the refractive index of the surrounding medium. Finally, we have *κ*^2^, which is a numerical factor that depends on the relative orientation of the two energetically coupled transition dipoles. From eqn 1, one can see that *E* = 1/2 precisely when *r* = *R*_0_ and thus the characteristic Förster distance defines the nanoscopic distance between the two fluorophores at which half of all donor relaxation events proceed via energy transfer to the acceptor [2].

After an energy transfer event, the newly excited acceptor can then de-excite via fluorescence, resulting in the emission of a photon (Figure 1C). Therefore, if one assumes that direct excitation of the acceptor is negligible [24], then photon emission from the acceptor requires FRET whereas photon emission from the donor precludes it. As such, the transfer efficiency, *E*, can be approximated via the amount of fluorescence from the acceptor (*F*_A_) and donor (*F*_D_) fluorophores (eqn 3).

(3) $$E=\frac{F_{A}}{F_{A}+F_{D}}$$

Furthermore, when important instrumental and experimental correction factors for spectral cross-talk and the nonidentical quantum yields and detection efficiencies of the two fluorophores are known [24–28], *F*_A_ and *F*_D_ can be used to more accurately determine the value of *E* enabling biochemists to quantify the nanoscopic distance, *r*, between the centers of the two fluorophores with sub-nanometer accuracy [29]. In this way, the neighboring donor and acceptor fluorophores of a FRET-labeled biomolecule function together as a spectroscopic ruler [30,31] for attaining nanoscale distances. Over the past half century, this experimental approach has allowed researchers to precisely measure the structural properties of fluorescently labeled molecules associated with a wide range of biochemical processes [31–34].

Many of the earliest FRET measurements took place in the cuvette of a standard ensemble fluorometer, with *F*_A_ and *F_D_* extracted from the resulting emission spectra. Such experiments are most informative when the sample contains a homogeneous population of molecules; however, in practice, this is rarely the case. Biological samples are often conformationally (e.g., folded/unfolded) and/or functionally (e.g., ligand-bound/free) heterogeneous. Furthermore, in FRET-based investigations, the fluorescent samples can also be compositionally heterogeneous due to incomplete labeling or inactive fluorophores. Unfortunately, the fluorescence from these various subpopulations cannot be well-resolved using a standard fluorometer, resulting in a single emission spectrum for the entire molecular ensemble, which in many cases can lead to inconclusive and sometimes even erroneous results.

Another limitation of these ensemble FRET measurements is that most time-dependent investigations occur out of equilibrium, where kinetic parameters must to be extracted from the system’s relatively gradual return to equilibrium [35,36]. Here, the molecular ensemble is synchronized using rapid equilibrium perturbation techniques (e.g., stopped-flow mixing), which imposes strict constraints on the temporal duration of the perturbation and/or the range of accessible time-scales. Fortunately, many of the complications arising from heterogeneous and asynchronous samples are largely the consequence of ensemble averaging, which can simply be avoided by studying single molecules.

## Single-molecule FRET

**Figure 2:**
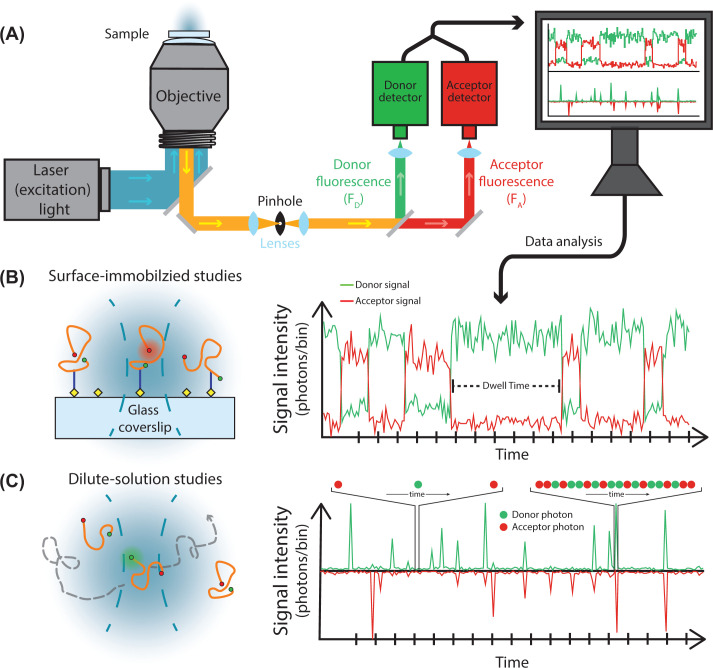
Two approaches to study single molecules using FRET and confocal fluorescence microscopy (**A**) General overview of a single-molecule confocal fluorescence microscope system, depicting the path of the excitation light (teal) as well as the path of the donor and acceptor fluorescence emission (solid red and green). (**B**) Generic graphical depiction highlighting one of several surface-immobilized approaches and the associated immobilization chemistry (yellow diamonds). Shown on the right is an artistic representation of donor and acceptor fluorescence time traces from a surface-immobilized biomolecule (orange). The horizontal length of the dashed line corresponds to the dwell time of a particular conformation. (**C**) Generic graphical depiction of the dilute-solution approach with single molecules transiently diffusing through the femtoliter observation volume (dashed hyperbola). The bursts of fluorescence from these transient events can be seen in the accompanying artistic representation of the fluorescence time trace on the right.

Given the extreme sensitivity required to detect single fluorophores, most single-molecule FRET measurements are carried out utilizing either confocal (Figure 2A) or wide-field fluorescence microscopes rather than a fluorometer. Here, excitation is generally accomplished using monochromatic light from lasers rather than polychromatic light from gas-discharge lamps. Importantly, the high numerical aperture objective of a microscope directs a much larger fraction of emitted photons towards the detection system when compared with the optical elements of a fluorometer, resulting in increased photon collection efficiencies. Unlike the precise spectral resolution provided by the monochromators of fluorometers, the donor and acceptor emission collected in single-molecule FRET experiments are generally resolved using a dichroic mirror and emission filters resulting in a spectrally broad band of fluorescence for each fluorophore. The spectrally resolved fluorescence from the acceptor (*F*_A_) and donor (*F*_D_) fluorophores is then recorded by sensitive cameras (wide-field microscopy) or photodiodes (confocal microscopy) with detection efficiencies that often exceed those associated with the photomultiplier tubes of conventional fluorometers [37].

As the name suggests, single-molecule FRET measurements require relatively low sample concentrations. In general, single-molecule conditions are most often achieved using one of two approaches. The first involves monitoring the fluorescence emitted from individual molecules sparsely immobilized to the surface of a passivated microscope coverslip (Figure 2B). The second involves recording the fluorescence emitted from a dilute solution containing picomolar concentrations of diffusing molecules (Figure 2C). Although the former can be accomplished using either wide-field or confocal microscopy, the latter is primarily restricted to confocal systems (Figure 2A).

Surface-immobilized studies exploit a wide range of biocompatible immobilization strategies [38–40] to sparsely affix FRET-labeled biomolecules, or liposomes encapsulating such molecules [41], to the surface of a microscope coverslip at a density of less than one molecule per μm^2^ [42]. The individual biomolecules are then exposed to the excitation light, ultimately resulting in the acquisition of donor and acceptor fluorescence time traces (Figure 2B), which are used to calculate *E* (via eqn 3) as a function of time. Not only do surface-immobilized studies provide structural information about the molecules of interest via the dipole–dipole distance-dependence of FRET, but these observations can also report on the dynamics and energetics of conformational equilibria that may be present. Depending on the experimental design and the type of microscopy employed, the duration of observation for each single molecule can range anywhere from milliseconds to minutes [43], with the maximum temporal resolution ultimately limited by the rate at which photons are detected. For molecular processes that occur on a time-scale much slower than the photon count rate, the conformational dynamics can readily be extracted from the anticorrelated fluctuations in the donor and acceptor fluorescence time traces [44,45]. Furthermore, the relative occupancy of these conformational subpopulations directly reports on the standard state free energy differences between them, providing additional biochemical insights into the process of interest. While surface-immobilized studies provide a wealth of structural, dynamic, and energetic information, one must consider the fact that surface immobilization is an additional experimental complication that needs to be carefully controlled because it can alter biomolecular behavior [46]*.*

Conveniently, the molecules of interest in dilute-solution studies are simply dissolved in solution at picomolar concentrations (Figure 2C). Under these conditions, the probability that more than one molecule resides in the femtoliter observation volume generated, in part, by the pinhole of a confocal microscope is frequently well below 10^−2^. This effectively ensures that any signal in the fluorescence time trace greater than that of the background is the result of a fluorescently labeled biomolecule transiently diffusing through the observation volume (Figure 2C). In these dilute-solution studies, the value of *E* for a single molecule is determined from the burst of donor and acceptor photons emitted during these transient events (Figure 2C). Although the duration of these events can vary greatly, the average diffusion time for a typical biomolecule is less than one millisecond and is determined by the molecule’s translational diffusion coefficient and thus its hydrodynamic radius. Given the sub-millisecond observation times of freely diffusing single molecules, these experiments are generally not well-suited for measuring conformational dynamics that occur on longer timescales. However, researchers have identified, and now routinely use, several different data analysis tools [47–60] or novel experimental approaches [61–63] to circumvent this limitation of dilute-solution studies. Conversely, dilute-solution studies are well-suited for studying molecular diffusion and other dynamic biochemical processes that occur on the micro-to-nanosecond timescales [64] via an approach called fluorescence correlation spectroscopy (FCS). Conceptually, this is accomplished by analyzing the frequency and amplitude of fluorescence intensity fluctuations resulting from translational diffusion or other sub-millisecond processes that cause fluctuations in the donor and/or acceptor fluorescence, including the conformational dynamics of a FRET-labeled biomolecule [65–68]. While the mathematical details underlying these correlation analyses are beyond the scope of this essay, they can be found elsewhere [2,65]. Nevertheless, it is still important to note that FCS is yet another useful spectroscopic tool that can be used to gain new insights into the conformational dynamics of FRET-labeled molecules.

At the most fundamental level, both dilute-solution and surface-immobilized studies report on biomolecular distances. This information is not unlike the spatial coordinates obtained from biomolecular simulations, which in some ways can simply be viewed as single-molecule distance measurements performed *in silico*. As such, several notable strategies have been developed during the last 10 years to combine the strengths of these two methods [24,69–85]. These strategies vary depending on the type of biochemical information desired. For example, molecular dynamics (MD) simulations use Newton’s equations of motion to generate time-dependent spatial coordinates of interacting atoms or particles and are most often used if the central research focus involves both biomolecular structure and dynamics. Alternatively, if structure and conformation are the primary focal points of the investigation, then information from various molecular modeling approaches can also be integrated with the data from single-molecule FRET experiments. In either case, single-molecule FRET results can be used to inform these computational approaches, which can then reinform the experimental design in an iterative process [86]. Although the details regarding the integration of these two approaches go beyond the scope of this brief introductory essay, they can be found in more advanced reviews on the topic [69,70,87–89]. However, the general idea is that the experimental data is used to either bias biomolecular simulations via structural constraints or as a means of validating specific computational models. Recently, integrative approaches involving single-molecule FRET and biomolecular simulations have been used to study the structural and dynamic aspects of several biochemical processes involving both proteins and nucleic acids [77–79,90–92].

## Nucleic acid–protein interactions

Macromolecular complexes comprised of proteins and nucleic acids are prevalent throughout all of biology and often carry out crucial biochemical tasks. Take, for example, the ribosome—a universal multi-megadalton RNA–protein construct responsible for protein synthesis [93]—or the various RNA and DNA polymerases whose precise and well-orchestrated interactions with the genetic material of biological entities are essential for life [94]. In fact, there is a diverse and seemingly limitless number of biochemical processes involving interactions between nucleic acids and proteins, many of which are not yet well-understood. In the following sections, we will highlight select research efforts from the past few years wherein integrative approaches utilizing both single-molecule FRET and biomolecular simulations were used to probe three different nucleic acid–protein interactions [90–92].

## RNA-folding chaperones and their nucleic acid clients

Proteins broadly known as ‘molecular chaperones’ are responsible for binding to and interacting with structured biomolecules in a way that facilitates the folding process and/or alleviates misfolding and aggregation [95]. A specialized class of chaperones known as RNA-folding chaperones are responsible for helping certain nucleic acids adopt these folded structures [96]. However, unlike many chaperones with protein clients, several RNA-folding chaperones are intrinsically disordered [91,96]. This notion of function sans structure is somewhat paradoxical, as many proteins must adopt well-defined structures to function properly [97,98].

**Figure 3:**
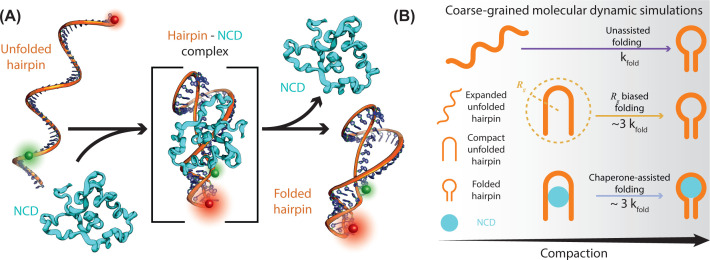
Combining single-molecule FRET and biomolecular computations to study RNA-folding chaperones (**A**) The nucleocapsid domain (NCD) of the hepatitis C virus core protein chaperones the folding of a FRET-labeled nucleic acid hairpin (**B**) Schematic representations of coarse-grained models used in molecular dynamic simulations. Simulations reveal that chaperone-induced compaction of unfolded nucleic acids increases the folding rate constant.

The nucleocapsid domain (NCD) of the hepatitis C virus core protein is one such example of an intrinsically disordered RNA-folding chaperone [99]. It is known to chaperone several conformational transitions in nucleic acids, including viral genome dimerization [100], which was recently shown to be important for viral replication [101]. To better understand the biochemical mechanisms governing this seemingly paradoxical behavior, Holmstrom et al. used single-molecule FRET and coarse-grained molecular dynamics to determine how this disordered protein influences the conformational dynamics of structured nucleic acids [91]. This was accomplished by labeling the 3′ and 5′ ends of a nucleic acid hairpin with donor and acceptor fluorophores (Figure 3A) in order to monitor folding and unfolding via changes in the transfer efficiency, *E.*

Holmstrom et al. showed how the chaperone alters hairpin folding by analyzing fluorescence time traces from surface-immobilized experiments conducted in the absence and presence of NCD*.* In the absence of NCD, they predominately observed low transfer efficiency values, indicative of an expanded and unfolded conformation, with infrequent and short-lived transitions to a higher transfer efficiency, indicative of a folded hairpin structure [91]. Importantly, the average dwell time of the two conformational states revealed that the unfolding rate constant was significantly faster than the folding rate constant. At saturating concentration of NCD, which were used to ensure that NCD is almost always bound to the hairpin, Holmstrom et al. observed an increase in the folding equilibrium constant with the hairpin more often occupying the high *E* conformation [91]. They also observed many short-lived transitions to an unfolded conformation that was significantly more compact than in the absence of NCD. The decreased average dwell time for the unfolded state suggests that the increase in folding equilibrium constant resulted almost exclusively from an increase in the folding rate constant. Holmstrom et al. hypothesized that the increased folding rate constant is directly related to the chaperone-induced compaction of the unfolded RNA [91], which effectively decreases the conformational search for the folded structure.

To test this hypothesis, the authors used several FRET-derived distance constraints from dilute-solution studies to optimize and validate a polymeric coarse-grained molecular model of both the hairpin and the chaperone. Here, each amino acid is represented by a single bead of the appropriate charge and each nucleotide represented by three beads—one for the sugar, phosphate, and base—with nonspecific interactions between the beads are governed by electrostatics and three short-range interactions depending on the macromolecular identity of the interacting beads. The MD simulations showed that chaperone binding both compacts the unfolded nucleic acid and increases the folding rate constant (Figure 3B). Importantly, the coarse-grained model of this interaction allowed the researchers to directly test the hypothesis that compaction of the unfolded state alone was sufficient to accelerate folding. Specifically, the dimensions of the free unfolded hairpin were computationally biased (via the radius of gyration) toward the more compact, yet still unfolded, conformations of the hairpin bound to the chaperone (Figure 3B). In the absence of NCD, the computationally enforced conformational bias was sufficient to produce a 3-fold enhancement of the folding rate constant that was comparable to the enhancement observed in unbiased simulations that included the chaperone, suggesting that the mechanism of NCD-assisted folding fundamentally relies on the compaction of the unfolded RNA [91].

## H1-mediated nucleosome compaction

**Figure 4:**
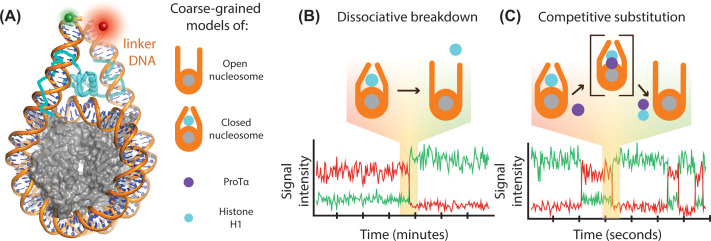
Combining single-molecule FRET and biomolecular computations to study H1-mediated nucleosome compaction (**A**) FRET-labeled linker DNA associated with the nucleosome-H1 complex. Artistic representations of fluorescence time traces highlighting H1 dissociating from the nucleosome in the absence (**B**) and presence (**C**) of prothymosin α (ProTα). Shown above each panel are schematic representation of coarse-grained models used to explain the enhanced dissociation of H1 via a competitive substitution mechanism.

Another class of highly disordered proteins are the so-called linker histones involved in chromatin compaction. Linear chromosomal DNA is wrapped around eight core histones forming a DNA–histone complex called the nucleosome. Linker histones interact with the nucleosome to help regulate the formation of condensed and higher order structures [102]. The human linker histone H1.0 (referred to as H1) is a positively charged protein with a small folded, globular core flanked by two highly disordered regions [103]. The disordered regions of H1 associate with the linker DNA (Figure 4A) forming condensed/compact nucleosomes [102].

Heidarsson et al. labeled the linker DNA with fluorophores to study the H1-induced compaction of nucleosomes using single-molecule FRET [90]. One key experimental finding from their surface-immobilized studies is that H1 dissociates from the nucleosome very slowly. At physiological ionic strength, they extrapolated that H1 would remain bound to the nucleosome complex for approximately an hour (Figure 4B), much longer than the minutes timescales observed *in vivo.* However, prothymosin α (ProTα) is a negatively charged intrinsically disordered protein and is thought to function as a linker histone chaperone that promotes dissociation of H1 from the nucleosome complex (Figure 4C). Upon addition of ProTα, the FRET-labeled nucleosome more frequently sampled low *E*, H1-unbound states, implying that the rate of H1 dissociation was substantially faster. Indeed, this change reveals that ProTα helps H1 dissociate from the nucleosome complex. The dependence on ProTα concentration also rules out simple first-order substitution mechanisms with a rate limiting step involving the dissociative breakdown of a binary H1–nucleocome complex. Therefore, in the presence of a linker histone chaperone like ProTα, the dissociation of H1 from the nucleosome is likely to proceed via a second-order competitive substitution mechanism involving the formation of a ternary complex with ProTα [90].

To further explore the formation of a transient ternary intermediate, Heidarsson and colleagues made 48 inter- and 12 intramolecular FRET measurements of H1, the nucleosome, and the complex they form. The results of these dilute-solution studies were then used to both optimize and validate a coarse-grained model of the three interacting species for MD simulations. Here, structure-based models of the nucleosome and the globular domain of H1 were used in conjunction with polymer-like representations of H1 and ProTα [84,85], where each amino acid is represented by single bead of the appropriate charge and each nucleotide represented by three beads. Importantly, the interaction between H1, ProTα, and the nucleosome were governed by a potential energy function containing electrostatic and generic short-range attractive terms, the latter of which was adjusted via a single parameter to maximize agreement with the experimental FRET data. When a polymeric model of ProTα was also included in the simulations, Heidarsson and colleagues observed that it would associate with the dynamic, disordered regions of the nucleosome-bound H1 [90]. Furthermore, they observed that it was much easier to displace H1 from the nucleosome with a computationally applied force when ProTα was part of the complex. Together these finding suggest that ProTα promotes H1 dissociation (and nucleosome expansion) via a competitive substitution mechanism involving the formation of a transient ternary intermediate (Figure 4C); a mechanism that the authors pose may be widespread among highly disordered polyelectrolyte complex [86].

## Architecture of the human telomerase holoenzyme

**Figure 5:**
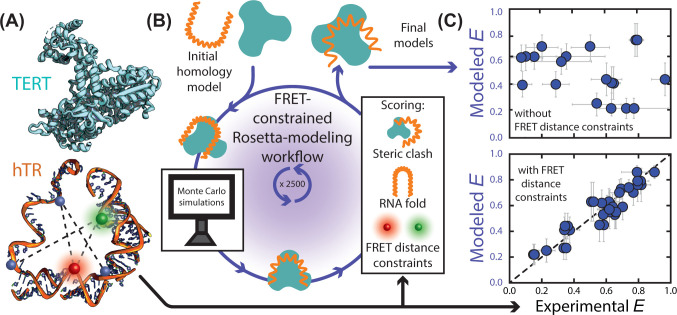
Combining single-molecule FRET and biomolecular computations to study the global architecture of the human telomerase holoenzyme (**A**) Structural models of the telomerase reverse transcriptase (TERT) and the human telomerase RNA (hTR) shown with multiple FRET-labeling position (colored spheres). (**B**) FRET-constrained Rosetta-modeling workflow for generating, scoring, and assessing the validity of modeled structures of the telomerase holoenzyme. (**C**) Agreement between experimental and modeled transfer efficiencies indicates that FRET-derived distance constraints are essential to accurately model the global architecture of the telomerase holoenzyme. Data reproduced with permission from [92] under a Creative Commons License.

Given the requirements associated with lagging strand replication, the ends of linear chromosomes, called telomeres, cannot be replicated [104]. This gives rise to the so-called ‘end-replication problem’ [105] wherein these linear chromosomes get progressively shorter with every replication cycle [106]. Telomerase consists of RNA-dependent DNA polymerase (TERT) and an essential RNA template (hTR) subunit (Figure 5A). This enzyme can alleviate the end-replication problem in highly replicative cells by processively elongating the telomeres to buffer against this inevitable loss of genetic material [107].

Prior to recently solving the structure of the entire telomerase enzyme [108–110], researchers lacked the necessary structural framework required to fully understand the functional mechanism of telomerase. Therefore, Parks and colleagues [92] employed a clever combination of single-molecule FRET and Rosetta-based structural modeling [111] to map out the global architecture of this important ribonucleoprotein complex. This integrative stochastic modeling approach involves simultaneously folding the human telomerase RNA (hTR) using fragment-based assembly [112] and docking it on to the surface of the TERT protein, using experimentally derived FRET data as flexible distance constraints.

In order to directly probe the global structure of the RNA subunit within the active holoenzyme, Parks et al*.* coupled donor and acceptor fluorophores to hTR using a total of five distinct labeling positions (Figure 5A) resulting in 10 different FRET-labeled constructs [92]. Then, they used surface-immobilized single-molecule FRET in conjunction with wide-field microscopy to record hundreds of fluorescence time traces for each of the 10 different hTR constructs in complex with TERT. This information allowed them to construct a network of distance constraints that were used to guide their stochastic modeling runs (Figure 5B). First, an initial homology model was constructed using several existing structures of small isolated RNA and protein domains of the telomerase enzyme. This molecular scaffold served as the initial starting point for each of the Rosetta modeling runs. The resulting 2500 models from each of the independent trials were assigned a Rosetta energy score based on three different metrics: an RNA folding score based on a library of experimentally determined RNA structures, an RNA–protein steric clash score based on ribonucleoprotein structures in the Protein Data Bank, and a FRET network score. Importantly, the inclusion of FRET-derived distance constraints was imperative for structural convergence of the independent trials, producing clusters or classes of well-scoring models. Furthermore, the near one-to-one correlation between experimental and model transfer efficiencies indicated that the converged models were consistent with the experimental data and therefore that the models provide an accurate representation of the global architecture of hTR within the context of the holoenzyme (Figure 5C). Notably, their FRET-constrained models of the human telomerase enzyme were structurally homologous to a concurrently published cryoEM-based structural model of the *Tetrahymena* enzyme, nicely showcasing the utility of this integrative approach.

With a robust structural model in hand, Parks et al. were then able to identify important conformational changes that occurred during the enzymatic cycle by comparing the modeled structure of the active complex to that of a nucleotide-starved (i.e., stalled) complex [92]. This led Parks et al. to propose a model wherein the global motion of a highly conserved pseudoknot domain within hTR contributes to the processivity of telomerase [92], enabling several repetitive cycles of telomere elongation to occur while the enzyme remains bound. Importantly, the work of Park et al. provides yet another example of an integrative combination of single-molecule FRET and computational simulations being used to extract structural insights from important biochemical processes.

## Conclusion

Single-molecule Förster resonance energy transfer is a versatile spectroscopic method for detailed investigations of many biochemical processes, including those involving interactions between nucleic acids and proteins. Importantly, the dipole–dipole distance-dependence of FRET makes it possible to monitor the nanoscale conformations of these biomolecules using fluorescence spectroscopy. Alone, these approaches can be used to provide detailed energetic and kinetic insights into the functional mechanisms of these molecules. However, when integrated with molecular dynamics, stochastic modeling, or other biomolecular simulation, the rich experimental data from single-molecule FRET can be used to unveil the mechanistic intricacies of a multitude of biological processes that are fundamental to life on Earth.

## Summary

- Single-molecule FRET is a highly informative fluorescence-based approach that can be used to measure various structural, energetic, and dynamic properties of biological polymers.
- The information acquired from these spectroscopic measurements can be readily integrated with biomolecular simulations to gain additional structural and/or mechanistic insights.
- Integrative approaches are being used to characterize a wide range of nucleic acid-protein interactions, yet many systems remain entirely unexplored and are ripe for investigation.

## Abbreviations

dNTP: deoxynucleoside triphosphate
FCS: fluorescence correlation spectroscopy
FRET: Förster resonance energy transfer
H1: human linker histone H1.0
hTR: human telomerase RNA
MD: molecular dynamics
NCD: nucleocapsid domain of the hepatitis C virus core protein
ProTα: prothymosin α
TERT: telomerase reverse transcriptase
